# CyberKnife therapy of 24 multiple brain metastases from lung cancer: A case report

**DOI:** 10.3892/ol.2013.1383

**Published:** 2013-06-07

**Authors:** GUIQING YANG, YISHAN WANG, YUANYUAN WANG, SIXIANG LIN, DONGNING SUN

**Affiliations:** 1 Center for Tumor Treatment, People’s Liberation Army 107th Hospital, Lai Shan Qu, Yantai, Shandong 264003, P.R. China; 2 Binzhou Medical College, Lai Shan Qu, Yantai, Shandong 264003, P.R. China

**Keywords:** brain tumor, CyberKnife, metastases, lung cancer

## Abstract

Brain metastasis is a significant cause of morbidity and mortality and a critical complication of non-central nervous system primary carcinoma. The present study describes the clinical case of a 46-year-old male with lung cancer and life-threatening brain metastases. The patient was diagnosed with lung cancer with a clinical stage of T2N0M1 (stage IV). Six months after the initial diagnosis and administration of conformal radiotherapy combined with three cycles of chemotherapy, an enhanced computed tomography (CT) scan of the brain revealed abnormalities with double-dosing of intravenous contrast. The CT scan identified >24 lesions scattered in the whole brain. The patient was treated with three-fraction Cyberknife radiotherapy at 22 Gy, delivered to the brain metastases at the Center for Tumor Treatment of People’s Liberation Army 107th Hospital. Following CyberKnife therapy, a CT scan of the brain revealed that most of the tumors had disappeared with almost no residual traces. The stereotactic radiosurgery (SRS) conducted using CyberKnife, an image-guided frameless robotic technology for whole-body radiosurgery, had produced a marked response. The present case report demonstrates that CyberKnife therapy plays a significant role in the management of multiple meta-static brain tumors.

## Introduction

Brain metastases are a common type of intracranial malignancy derived from the transfer of tumor cells outside the central nervous system to the brain tissue. In a study by Lu-Emerson and Eichler, brain metastases were reported to occur in ∼20–40% of cancer patients, with an onset age of 50 to 70-years-old. The study reported that 1/3–1/2 of cancer patients died as a direct result of brain metastases (1). The clinical manifestations of brain metastasis often insidiously develop within a few weeks, with only 1/4 of patients developing manifestations suddenly. Whole brain radiation therapy (WBRT) is suitable for multiple brain metastases, and a previous study has shown that 80% of patients who undergo WBRT experience a good therapeutic effect. The natural course of untreated multiple brain metastases is only one month and the median survival after whole brain irradiation is 4–6 months (2). The surgery is mainly administered to patients with single brain metastases and good bodily functions. In recent years, surgery has been gradually replaced by stereotactic radiosurgery (SRS). SRS is able to accurately determine the spatial location of the target metastases and precisely converge high-energy radiation to the target tissue, with almost no effect on the surroundings. Focused, highly-targeted irradiation and the delivery of fractionated CyberKnife therapy is considered to be an effective treatment for large multiple brain metastatic tumors. Written informed consent was obtained from the patient.

## Case report

A 46-year old male smoker was admitted to the Center for Tumor Treatment of the People’s Liberation Army 107th Hospital (Yantai, Shandong, China) in April 2010. The patient was suffering with a long-term cough and pain in the chest and back. Using X-ray, a mass was detected in the right lobe of the lung. Enhanced computed tomography (CT) scans confirmed the suspicion of lung cancer and also identified a right adrenal metastasis. The tumor was clinically staged as T2N0M1 (stage IV). The patient underwent conformal radiotherapy with 66 Gy/33 F combined with three cycles of cisplatin and vinorelbine chemotherapy. A good partial remission was achieved after the administration.

**Figure 1.**
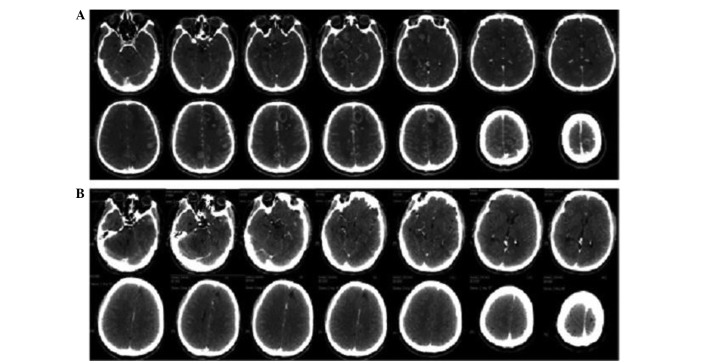
Multiple intracranial metastases (24 lesions). Computed tomography (CT) revealed that the tumors had disappeared following CyberKnife treatment. (A) CT prior to CyberKnife therapy (2010-12-04); (B) CT following CyberKnife therapy (2011-02-22).

Six months after the initial diagnosis, the patient experienced severe headaches with frequent vomiting and blurred vision. An enhanced CT scan of the brain revealed abnormalities with a double-dosing of intravenous contrast, which demonstrated that there were no less than 24 lesions scattered in the whole brain, including the frontal, parietal and temporal lobes and the brainstem (Fig. 1A). A discussion was held by experts in the field with regard to the multiple brain metastases, which were highly suspicious and stemmed from the primary lung cancer, and surgical treatment was consequently rejected. The patient received treatment using the CyberKnife Robotic Radiosurgery System on December 4, 2010, using an 80% prescription dose line covering 95% of the planning target volume. A total of 22 Gy was delivered in 3 fractions to the brain metastases. The modified conformity index was 1.41. During regular follow-up examinations, the headache symptoms were found to be markedly alleviated, the vomiting and blurred vision were slowly eased and there were no side effects from the radiation. The tumor shrank within 1 month and had completely disappeared within 3 months (Fig. 1B). The patient succumbed to hepatic metastases 1 year after the CyberKnife therapy.

## Discussion

Brain metastases from systemic cancer are the most common type of intracranial neoplasm in adults, being almost 10 times more common than primary malignant brain tumors, which cause a significant burden on the management of patients with advanced cancer (1). The lungs represent one of the most frequent sources of metastases to the brain, with a probability of (36–64%) (3). Symptoms suffered by the patients include headaches, epilepsy, focal weakness, numbness or changes in mental status. The prognosis of patients with brain metastases is not optimistic and the median survival time is ∼1–2 months if left untreated. The 1-year survival rate has been recorded as 10.4% (4,5). The treatment of metastatic brain tumors is complex; not only due to being able to provide local control and improve neurological function, but also due to factors such as age, performance and systemic disease status and the size, volume, location and number of metastases at presentation (1). There have been a number of studies describing metastatic brain tumors being successfully eliminated by surgery, chemotherapy, WBRT or a combination of modalities (6,7). These modalities have prolonged the survival time of patients and improved the neurological outcome. Craniotomy, which allows immediate relief of symptoms of intracranial hypertension, a reduction of focal neurological deficits and seizures, or use of a rapid steroid taper, has become the preferred therapy for metastatic brain tumors (8). The majority of studies support surgery as a suitable treatment for patients with single brain metastases with a mean maximum diameter of 3.43±0.74 cm, but not for patients with multiple brain metastases due to more frequent leptomeningeal dissemination following craniotomy (9,10). Furthermore, the mortality rate of patients who undergo surgery for brain metastases is ∼2- to 4-fold higher than that of patients with primary brain tumors (11).

WBRT has been widely used for the management of brain metastases, particularly multiple brain metastases, for decades, as it results in the rapid improvement of neurological symptoms and prolongs survival (12). However, radiation- induced dementia is a well-known side effect, although it does increase the median survival time to 4–6 months (2). SRS, particularly γ-Knife surgery (GKS), affords excellent local tumor control for between 1 and 10 brain metastases (13,14). Grandhi *et al* (15) assessed the clinical outcomes of 61 patients with ≥10 brain metastases who underwent SRS at the Leksell γ-Knife Perfexion (LGK PFX) unit (University of Pittsburgh Cancer Institute, Pittsburg) for the treatment of 806 tumors (mean, 13.2 lesions). Of the total treated tumor volume, actuarial freedom from local tumor progression was 94.1% at 3 months after LGK PFX surgery, 90.5% at 6 months and 58.3% at 12 months. It appeared that SRS was conducive to a prompt response and good outcome. However, SRS has not been applied easily for multiple large brain metastases due to serious medical conditions, with increased intracranial pressure or insufficient marginal dose, although it has been accepted as a general treatment method for brain metastasis since 1975 (16).

CyberKnife is a robotic radiosurgery system with a linear particle accelerator (linac), which is coupled with real-time imaging to track and compensate for the patient’s or target’s motion. As a relatively non-invasive treatment modality, CyberKnife demonstrates certain benefits, including a more accurate target localization and improved dose delivery for the management of metastatic brain tumors that allows higher biologically effective dose delivery without increased incidence of toxicity. Nishizaki *et al* (17) produced a retrospective study on 71 patients with 148 metastatic brain lesions who underwent CyberKnife therapy. A total of 31 patients had multiple lesions (range, 2–7) at the initial treatment, and 86 lesions achieved local control. The median survival time was 56 weeks. Overall, 40 patients succumbed to progressive brain metastases, primary cancers, extracranial metastases and other diseases, including infection or gastric bleeding. No patient succumbed as a result of intracranial disease from new metastases.

However, few studies have demonstrated a marked change in the typical cohort of patients with 1 to ≥20 brain metastases. In the present case, the 24 brain metastases (originating from lung cancer), which were scattered throughout the whole of the brain, were completely resolved within 3 months following CyberKnife radiosurgery. No opportunistic recurrence or metastasis occurred during the follow-up, as revealed by CT imaging studies. Although the patient succumbed to liver metastasis, the survival results were comparable to those of published studies. Kim *et al* (18) conducted a study in 26 patients with ≥10 brain metastases; following GKS the median survival was 34 weeks. Grandhi *et al* (15) showed that the median survival time of patients with ≥14 brain metastases after LGK PFX treatment was 3 months compared to 6 months in those with <14 lesions. The survival time of the present study is similar to that cited in the study by Nishiazki *et al,* which revealed a survival time of ∼56 weeks when the patients were treated with CyberKnife (17).

In the present case, the results for the treatment of multiple brain metastases after CyberKnife surgery with a 7–8 Gy marginal dose was promising. CyberKnife for metastatic brain tumors is an effective and safe method for reducing the marginal dose prescribed for multiple brain metastases and for minimizing the radiation-related neurotoxicities. In conclusion, CyberKnife, a focused, highly-targeted radiosurgery and fractionated radiotherapy is particularly useful for multiple brain metastases. CyberKnife provides the advantage of the management of local recurrence and a tolerable complication rate. Although the treatment of brain metastases has been performed with CyberKnife, the clinical significance and optimal dose fractionation scheme require further investigation.
